# Gabapentin improves neuropathic pain in Minamata disease model rats

**DOI:** 10.1265/ehpm.24-00035

**Published:** 2024-05-31

**Authors:** Masatake Fujimura

**Affiliations:** Department of Basic Medical Sciences, National Institute for Minamata Disease, Minamata, Japan

**Keywords:** Methylmercury, Minamata disease, Neuropathic pain, Gabapentin, Somatosensory cortex, Synaptic rewiring

## Abstract

**Background:**

Methylmercury (MeHg), the causative agent of Minamata disease, damages the cranial nervous system and causes specific sensory disturbances, especially hypoesthesia, in the extremities. However, recent reports demonstrate that patients with chronic Minamata disease conversely develop neuropathic pain in the lower extremities. Studies on our established Minamata disease model rats showed that MeHg-mediated neurodegeneration might induce neuropathic pain by over time through inducing rewiring with neuronal activation in the somatosensory cortex via microglial activation in the spinal dorsal horn.

**Methods:**

In this study, the effects of gabapentin, a potentially effective treatment for neuropathic pain, was evaluated using this Minamata disease model rats. To further elucidate the mechanism of its medicinal effects, histochemical and biochemical analyses of the nervous system of Minamata disease model rats were conducted.

**Results:**

Gabapentin treatment restored the reduction in the pain threshold caused by MeHg exposure in rats. Histochemical and biochemical analyses revealed that gabapentin showed no effect on MeHg-induced neurodegeneration in entire nervous system and microglial activation in the spinal dorsal horn. However, it was shown that gabapentin may reduce excessive synaptogenesis through its antagonist action on the alpha2-delta-1 subunit of calcium channels in the somatosensory cortex.

**Conclusions:**

These results indicate that gabapentin may alleviated neuropathic pain in MeHg poisoning, as typified by Minamata disease, by reversibly modulation synaptic rewiring in the somatosensory cortex.

**Supplementary information:**

The online version contains supplementary material available at https://doi.org/10.1265/ehpm.24-00035.

## Background

Methylmercury (MeHg) is a heavy metal ubiquitous in nature and routinely ingested by people who eat seafood. However, ingesting food contaminated by large amounts of MeHg can cause serious neurological damage. MeHg poisoning was caused by the ingestion of contaminated fish in Japan [1], where it is referred to as Minamata disease, and by ingesting contaminated seeds in Iraq [2]. The acute phase of MeHg poisoning results in neurosensory dysfunction, including balance, visual, auditory, and somatosensory disturbances caused by nerve damage to the central and peripheral nerves such cerebral cortex, cerebellum and dorsal root nerve [3–5]. These neuropathological changes can also be reproduced in rodent models of MeHg poisoning [6].

Neuropathic pain is reportedly observed in the lower extremities of patients with chronic Minamata disease [7, 8]. Neuropathic pain from nerve injuries other than diabetes and herpes is likely due to either peripheral nerve-spinal cord injury or blockage of the descending inhibitory pathways by thalamic injury, etc. [9–12]; both types of nerve injury are thought to cause neuropathic pain by stimulating the somatosensory cortex through neuronal activation in the dorsal horn of the spinal cord [13–15]. In my previous studies, a model of neuropathic pain with MeHg exposure has been established using rats [16, 17]. This rat model has been confirmed that MeHg exposure caused neuropathic pain by damaging peripheral nerve-spinal cord and thalamic nerves.

Gabapentin was first approved in the United Kingdom and the United States as an adjunctive therapy for partial-onset seizures in adults with epilepsy [18]. After approval for pediatric indications in the European Union and the United States in 1999, gabapentin has demonstrated global applications as an antiepileptic drug, including in Asia. Gabapentin is thought to achieve antiepileptic effects by inhibiting Ca channels, which are Ca^2+^ pathways in the presynapses of excitatory glutamatergic nerves, inhibiting the release of excitatory neurotransmitters by suppressing Ca^2+^ influx, and increasing the amount of GABA, an inhibitory neurotransmitter in the brain [19, 20]. Subsequently, gabapentin was shown to be highly useful and effective for treating neuropathic pain [21, 22] and is now widely used as a treatment for this disease. Although some researchers have suggested that the mechanism of action of gabapentin for neuropathic pain involves inhibition of neural activity in the spinal cord pathway [23–25], no definitive proof has been obtained. Other studies have demonstrated that gabapentin acts on a receptor for the alpha 2 delta-1 subunit of calcium channels, which is responsible for excitatory central nervous system synaptogenesis [26, 27]. Currently, the action of gabapentin on this receptor, which antagonizes thrombospondin (TSP)-induced synaptic crosstalk in the somatosensory cortex, has been speculated to be one of the primary mechanisms by which gabapentin inhibits neuropathic pain [28, 29].

Our previous studies have revealed that pain in Minamata disease model rats, similar to common neuropathic pain, is caused by damage to the dorsal root nerve and thalamus. Therefore, the hypothesis arises that gabapentin might also improve MeHg-induced neuropathic pain. This study investigates whether gabapentin is effective against neuropathic pain in Minamata disease model rats and explore the underlying mechanism of action.

## Methods

### Animals and experimental schedule

Thirty-two 6-week-old male Sprague-Dawley rats (230–260 g body weight) were purchased from CLEA Japan (Tokyo, Japan) and acclimated for one week. All animals were kept ad libitum for food and water in plastic cages (width 247 × depth 355 × height 198 mm) containing 2–3 animals each in a breeding room maintained at 24 °C, humidity 50∼70%, and a 12 h light/dark cycle. First, 8 rats were treated with the vehicle (glutathione only) and the remaining 24 were treated with 20 ppm of MeHg (Tokyo Chemical Industry Co., Ltd., Tokyo, Japan) in the form of MeHg-glutathione complex, as previously described [30], in drinking water for three weeks. This MeHg dose in drinking water is equivalent to 1.0 mg/kg/day [31]. After an additional three-week withdrawal period, the 24 MeHg-treated rats were divided into three groups of eight rats each, and each group received either the vehicle (ultrapure water only), low-dose gabapentin (Tokyo Chemical Industry Co., Ltd., 800 ppm in drinking water), or high-dose gabapentin (1,200 ppm in drinking water) for three weeks; these gabapentin doses in drinking water are equivalent to 40 or 60 mg/kg/day, respectively. After completion of drug administration, all animals were dissected in deep anesthesia by isoflurane inhalation (Abbot Japan, Tokyo, Japan) and subjected to biochemical and histopathology experiments. The experimental conditions of the Sup. Fig. are described in a previously reported paper [16].

All animal experiments were conducted in accordance with the “Guide for the Care and Use of Laboratory Animals” stipulated by the National Institute for Minamata Disease. This guidance is in accordance with the approved by the Institutional Animal Care and Use Committee (IACUC).

### Measurement of the mechanical pain threshold

Mechanical pain thresholds were assessed once per week until drug administration was completed. Mechanical pain thresholds were measured in the plantar region of rat hindlimb using a rodent pincher-analgesia meter (Bioseb, Pinellas Park, FL, USA), as previously described [16, 17]. The pain threshold was measured three times at 5 s intervals for one individual. The threshold for each individual was calculated by averaging the three values.

### Histopathological analyses

Neuropathological analysis was performed as previously described [17, 32]. Paraffin-embedded sections were prepared from the dorsal root nerve and spinal cord, and stained with hematoxylin and eosin (Dako Corporation, Carpinteria, CA, USA). The paraffin sections of the brain were immunohistochemically stained using NeuN (Chemicon, Temecula, CA, USA) according to previously described procedures, and quantitatively analyzed for the number of neurons in the somatosensory cortex and thalamus [17, 33]. Furthermore, paraffin sections of the spinal cord were immunohistochemically stained using Iba1 (FUJIFILM Wako Pure Chemicals, Osaka, Japan) for quantitative analysis of Iba1-positive microglia as an indicator of microglial activation [34] in the dorsal horn of the L3 spinal cord [16, 17].

For the Sup. Fig. experiment, skin sections from the center of the plantar part of the hind paw were stained with hematoxylin and eosin.

### Biochemical analyses

The isolated and collected L3 posterior spinal cord and somatosensory cortex were subjected to Western blotting analysis using a previously described method [35–37]. In this study, antibodies against the following proteins were used: inducible nitric oxide synthase (iNOS), interleukin (IL)-1β, IL-6, arginase-1, IL-10, β-actin, nuclear factor kappa-light-chain-enhancer of activated B (NFκB p65), phospho-NFκB (Ser-536), CRE binding protein (CREB), phospho-CREB (Ser-133), brain-derived neurotrophic factor (BDNF), postsynaptic density protein 95 (PSD95) (all from Cell Signaling Technology, Danvers, MA, USA), tumor necrosis factor-alpha (TNF-α), and vesicular glutamate transporter (vGlut) 1 (GeneTex International Corporation, Hsinchu City, Taiwan), TSP-1 (Thermo Fisher Scientific, Waltham, MA, USA). The concentration of each protein was standardized related to β-actin or its total protein.

### Measurement of tissue mercury concentration

Total mercury concentrations in the somatosensory cortex, thalamus, and L3 spinal cord were measured using a heating vaporization method [38] with a mercury analyzer (MA2000; Nippon Instruments, Tokyo, Japan).

### Statistical analysis

All data were subjected to one-way ANOVA followed by Dunnett’s multiple comparison test. In this study, statistical significance was determined at p < 0.05 or greater.

## Results

### Effect of gabapentin on neuropathic pain in Minamata disease model rats

**Fig. 1:**
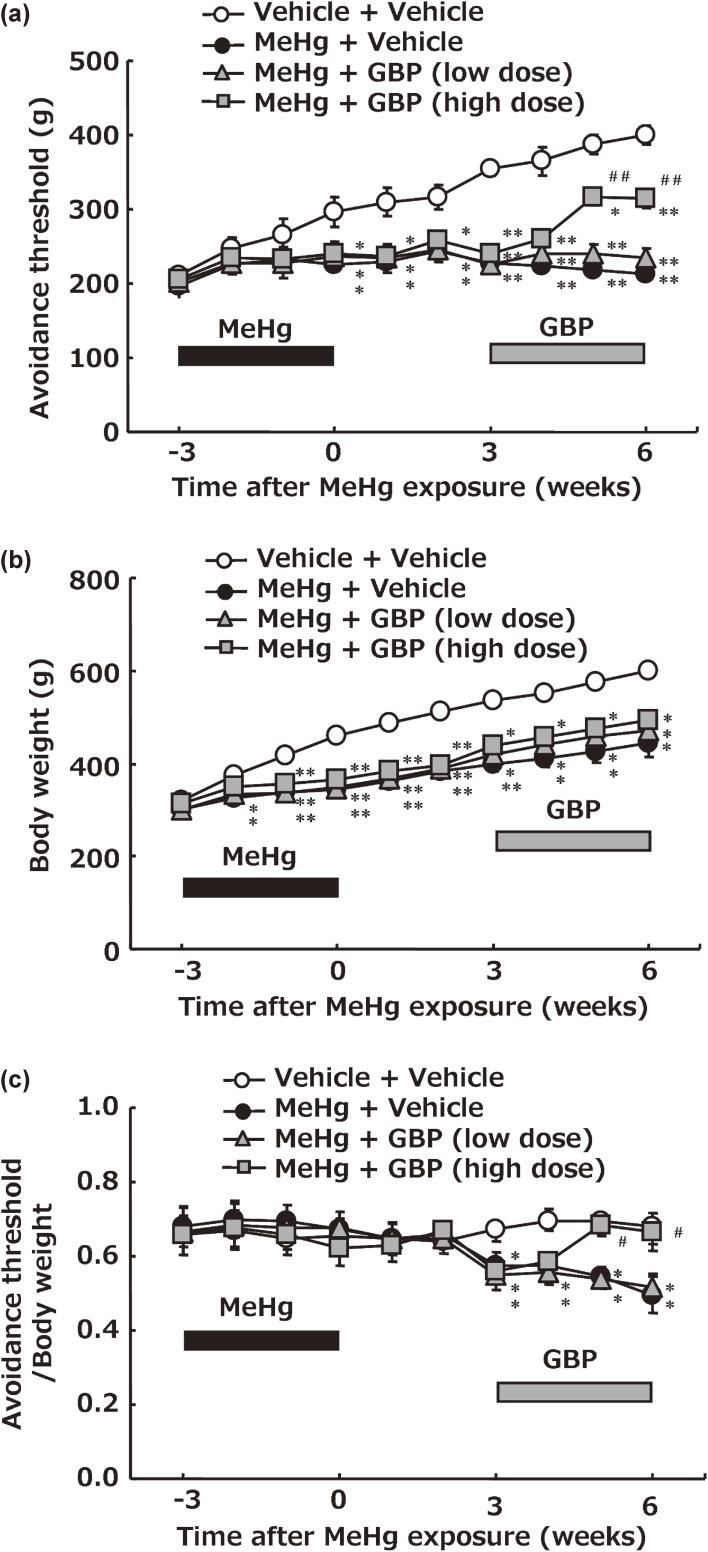
Time-course change in mechanical pain threshold in Minamata disease model rats. Values represent the mean ± SEM (n = 8). Significant differences were observed between the vehicle + vehicle-treatment group and MeHg treatment groups (^*^p < 0.05 and ^**^p < 0.01) and between the MeHg + vehicle-treatment group and MeHg + gabapentin-treatment groups (^#^ p < 0.05 and ^##^ p < 0.01). (a) Time-course change in mechanical pain threshold. (b) Body weight. (c) Mechanical pain threshold/body weight. GBP: gabapentin.

The mechanical pain threshold progressively increased with age in the control vehicle rats, whereas the MeHg-treated group did not show such an increase (Fig. 1a). Since an increase in mechanical pain threshold in the control vehicle group may be due to body growth, it was corrected for the influence of body growth. Body weight increased with age in the control group, whereas the weight gain was significantly suppressed in all MeHg-exposed rats (Fig. 1b). The mechanical pain threshold corrected by the influence of body weight is shown in Fig. 1c. The results showed that the mechanical pain threshold significantly decreased in MeHg-treated rats compared to that of the control group after 3 weeks of MeHg withdrawal, similar to the results of previous studies [16, 17].

Continuous administration of low-dose or high-dose gabapentin (800 ppm or 1,200 ppm in drinking water, respectively) was performed for three weeks after MeHg withdrawal. Gabapentin had no effect at low doses but induced significantly higher pain thresholds than those in untreated MeHg-exposed rats from 2 weeks of administration at high doses (Fig. 1c).

### Effect of gabapentin on histopathological changes in Minamata disease model rats

**Fig. 2:**
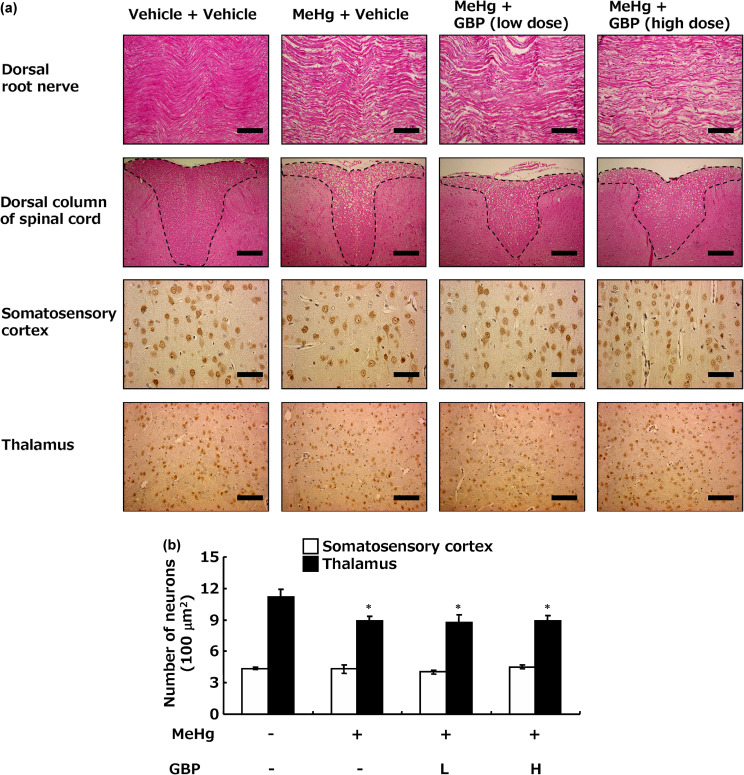
Histopathological analysis of Minamata disease model rats. Each image shows a representative image. (a) Hematoxylin and eosin staining in L3 dorsal root nerves (upper panel, scale bar = 100 µm) and the dorsal column of the spinal cord (within the dashed line is the dorsal column; second panel, scale bar = 200 µm), and immunohistochemical staining with anti-NeuN in the somatosensory cortex (third panel, scale bar = 50 µm) and thalamus (lower panel, scale bar = 100 µm). (b) Quantitative analysis of NeuN-positive neurons in the somatosensory cortex and thalamus. Each value was measured over the entire area of the coronal section of the thalamus and somatosensory cortex and expressed as the number of NeuN-positive neurons per 100 µm^2^. Values represent the mean ± SEM (n = 8). Significant differences were observed between the vehicle + vehicle-treatment group and MeHg treatment groups (^*^p < 0.05). GBP: gabapentin, L: low dose, H: high dose.

MeHg exposure showed irregular axonal organization and axonal disruption in the dorsal root nerves (Fig. 2a, upper panel), and vacuolization due to axonal disruption in the dorsal column of the spinal cord (Fig. 2a, second panel). Gabapentin administration did not repair these axons, even at the high dose that corrected the pain threshold. Furthermore, immunohistochemical analysis of anti-NeuN, a marker of mature neurons, was used to examine the effects of MeHg exposure on neuronal cell numbers. MeHg exposure had no effect on neurons in the somatosensory cortex but reduced the number of neurons in the thalamus (Fig. 2a, third and lower panel, 2b). Gabapentin administration did not affect neuronal cell numbers even at a high dose, similar to its effects on axons.

For the pathological analysis at the center of the plantar part of the hind paw, where the actual mechanical pain threshold be measured, the animal tissue from the previously reported paper [16] was used. The distance from the skin surface to the border between the epidermis and dermis, where non-nociceptive sensory receptors are present [39, 40], increased gradually with age in the control vehicle rats as well as with body weight, but no such increase was observed in the MeHg-treated group (Sup. Fig. 1a–c).

### Effect of gabapentin on microglial activation in Minamata disease model rats

**Fig. 3:**
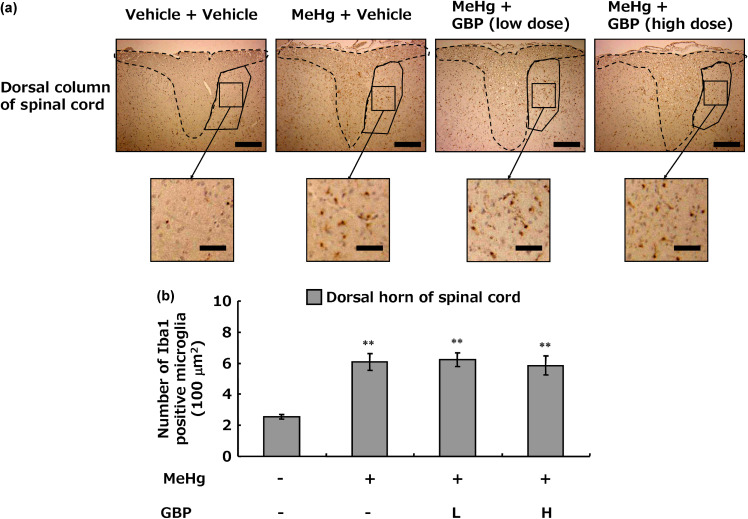
Activated microglia expression in the dorsal horn of Minamata disease model rats. Each image is a representative image. (a) Immunohistochemical staining with anti-Iba1 in the dorsal region of the L3 spinal cord (scale bar = 200 µm) and enlarged view of the dorsal horn (scale bar = 50 µm). Within the dashed line and solid line are the dorsal column and dorsal horn of the spinal cord, respectively. (b) Quantitative analysis of Iba1-positive microglia in the dorsal horn of the L3 spinal cord. Each value was measured over the entire area of the coronal section of the dorsal horn and expressed as the number of Iba1-positive microglia per 100 µm^2^. Values represent the mean ± SEM (n = 8). Significant differences were observed between the vehicle + vehicle-treatment group and MeHg treatment groups (^**^p < 0.01). GBP: gabapentin, L: low dose, H: high dose.

Immunohistochemistry for Iba1, a marker of activated microglia, was used to examine microglial changes in the dorsal horn of the spinal cord involved in neuropathic pain. MeHg exposure markedly increased the number of activated microglia in the dorsal horn of the spinal cord (Fig. 3a, b). However, gabapentin did not affect the number of activated microglia in the dorsal horn of the spinal cord, even at high doses (Fig. 3a, b).

### Effect of gabapentin on microglial phenotype changes, synaptic rewiring, and neuronal activation in the spinal cord including the dorsal horn of Minamata disease model rats

**Fig. 4:**
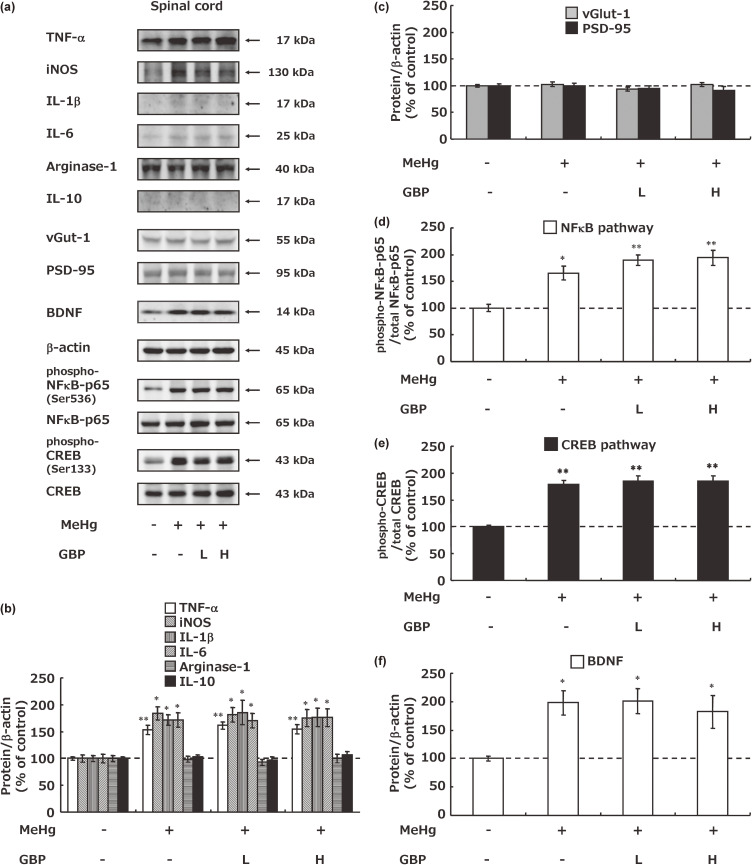
Microglia phenotype and neuronal activation in the spinal cord of Minamata disease model rats. (a) Western blotting analysis of microglia-derived cytokines and neuronal activation and their associated factors. The concentration of each protein was standardized using β-actin (b) or its total protein (c, d). Each value is expressed as the percentage compared to vehicle + vehicle-treated group. Values represent the mean ± SEM (n = 8). Significant differences were observed between the vehicle + vehicle-treatment group and MeHg treatment groups (^*^p < 0.05 and ^**^p < 0.01). GBP: gabapentin, L: low dose, H: high dose.

Using the Western blotting method, cytokines and related pathways involved in microglial phenotypes in the spinal cord including the dorsal horn was investigated. MeHg exposure increased the expression of pro-inflammatory cytokines such as TNF-α, iNOS, IL-1β, IL-6, and phosphorylated NFκB p65, but did not affect the expression of the anti-inflammatory cytokines arginase-1 and IL-10 (Fig. 4a, b, c). On the other hand, MeHg had no effect on synaptic rewiring in this region (Fig. 4a, c). Gabapentin did not affect the increase in proinflammatory cytokines and phosphorylated NFκB p65 induced by MeHg exposure. In addition, phosphorylated CREB and BDNF, markers of neuronal activation were measured. MeHg exposure induced neuronal activation, but gabapentin did not significantly inhibit this activation (Fig. 4a, e, f).

### Effects of gabapentin on synaptic rewiring and neuronal activation in the somatosensory cortex of Minamata disease model rats

**Fig. 5:**
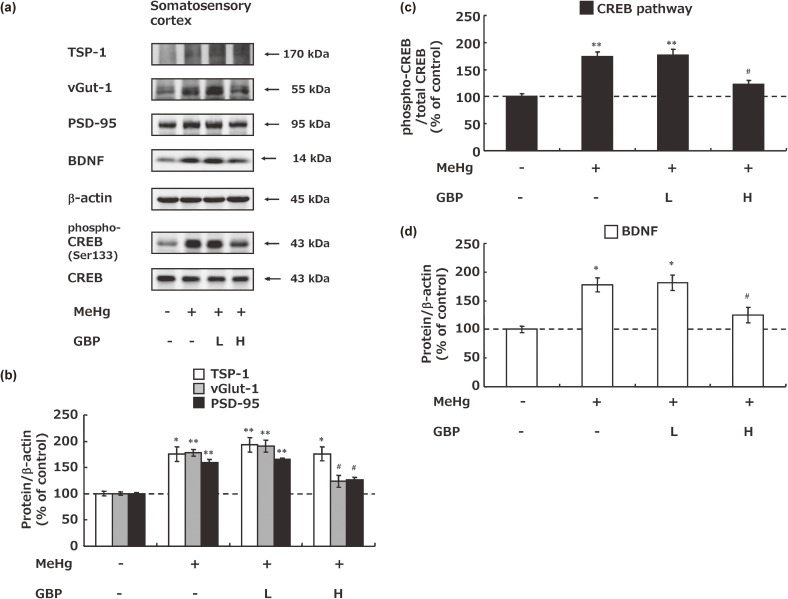
Synaptic rewiring and neuronal activation in the somatosensory cortex of Minamata disease model rats. (a) Western blotting analysis of factors involved in synaptic rewiring and neuronal activation. The concentration of each protein was standardized using β-actin (b) or its total protein (c). Each value is expressed as the percentage compared to vehicle + vehicle-treated group. Values represent the mean ± SEM (n = 8). Significant differences were observed between the vehicle + vehicle-treatment group and MeHg-treatment groups (^*^p < 0.05 and ^**^p < 0.01) and between the MeHg + vehicle-treatment group and MeHg + gabapentin treatment groups (^#^ p < 0.05). GBP: gabapentin, L: low dose, H: high dose.

The expression changes of proteins involved in synaptic rewiring in the somatosensory cortex were also investigated. MeHg exposure increased the expression of TSP-1 and synaptic markers such as vGlut-1 and PSD-95 in the somatosensory cortex (Fig. 5a). A high dose of gabapentin did not suppress the increase in TSP-1 expression but did significantly suppress the increase in the expression of synaptic markers such as vGlut-1 and PSD-95 (Fig. 5a, b). MeHg exposure activated neural activity, which was significantly suppressed by a high dose of gabapentin (Fig. 5a, c, d).

### Mercury concentrations in the somatosensory cortex, thalamus, and spinal cord including the dorsal horn of Minamata disease model rats

**Fig. 6:**
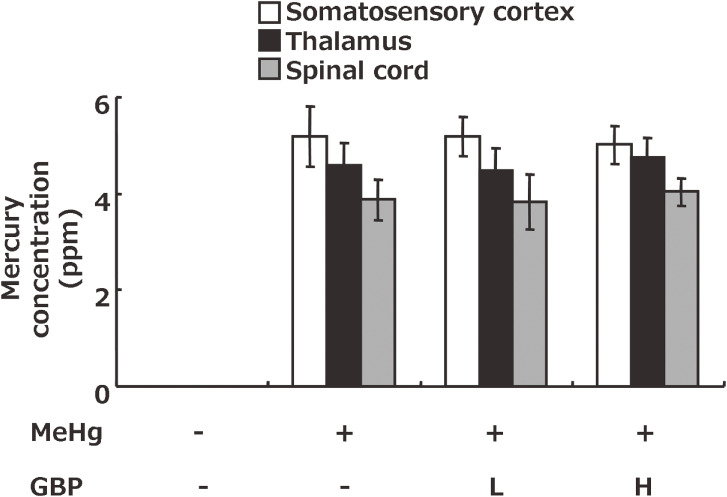
Mercury concentration in the somatosensory cortex, thalamus, and spinal cord of Minamata disease model rats. Values represent the mean ± SEM (n = 8). GBP: gabapentin, L: low dose, H: high dose.

MeHg exposure increased total mercury concentrations in the somatosensory cortex thalamus, and spinal cord including the dorsal horn. Gabapentin administration did not affect total mercury concentrations in either area (Fig. 6).

## Discussion

In this study, the improvement effect of gabapentin on neuropathic pain in Minamata disease model rats was demonstrated for the first time. Moreover, the results suggest that the mechanism of action involves a reversible modulation of synaptic rewiring in the somatosensory cortex rather than inhibition of microglial activation in the dorsal horn of the spinal cord.

Pain measurement was not straightforward. This was because in experiments with laboratory animals, the mechanical pain threshold progressively increased with age. Since the cause might be attributed to body growth, the effect of body growth was corrected for body weight, and the constant values (avoidance threshold/body weight: 0.6∼0.7) were successfully obtained. On the other hand, when the MeHg group was corrected for body weight, the corrected value decreased (avoidance threshold/body weight: around 0.5), suggesting that MeHg exposure causes neuropathic pain (Fig. 1). Furthermore, to determine the cause of the growth-induced increase in pain threshold, using animal tissue from a previously published paper [16], pathological examination at the center of the plantar part of the hind paw, where the actual measurement site of mechanical pain threshold. The distance from the skin surface to the border between the epidermis and dermis, where non-nociceptors reside [39, 40] gradually increased with age in control vehicle rats, but no such increase was observed in the MeHg-treated group (Sup. Fig. 1). These findings suggested that the growth-induced increase in pain threshold is due to plantar growth.

I clarified the effect of gabapentin, a drug that is expected to reduce the pain, by behavioral experiments using Minamata Disease model rats (Fig. 1). High-dose gabapentin (equivalent to 1.5 times the upper clinical dose limit of 40 mg/kg/day) [41] restored the reduction in the mechanical pain threshold induced by MeHg poisoning in rats, whereas the low dose of gabapentin (equivalent to 40 mg/kg/day, the upper clinical dose limit) showed no restorative effect on neuropathic pain. Once altered, the synaptic configuration was difficult to correct, suggesting that a higher dose of gabapentin was necessary for recovery from neuropathic pain. Therefore, it was considered that high-dose gabapentin is necessary for treatment after lowering the pain threshold in MeHg-poisoning.

Theoretical considerations are also necessary to bridge the species differences between humans and experimental animals with respect to pharmacological effects. Therefore, histological and biochemical methods were used to elucidate the mechanism of the reducing effect of gabapentin on pain. First the effects of gabapentin on damage to the dorsal root, spinal cord, and thalamic nerves that caused neuropathic pain was examined. Although MeHg-induced axonal damage to the dorsal root nerves and loss of thalamic neurons was confirmed, gabapentin did not improve the observed nerve damage (Fig. 2). As numerous studies have shown that microglial activation in the dorsal horn of the spinal cord play an important role in the initiation and maintenance of neuropathic pain after dorsal root nerve injury [10, 13, 42], microglial activation in the dorsal horn of the spinal cord in Minamata disease model rats was examined. Despite its effect on MeHg-induced neuropathic pain, a high dose of gabapentin did not prevent an increase in the number of Iba1-positive microglia in the spinal dorsal horn (Fig. 3). Furthermore, cytokines, environmental agents, and various physiological environments are known to shift microglia into a pro-inflammatory M1 or anti-inflammatory M2 state [43–45]. Pro-inflammatory cytokines and related factors, including TNF-α, IL-1β, IL-6, and nitric oxide, are produced/released from microglia of the M1 phenotype and subsequently activate the NFκB pathway [46–48]. Conversely, the anti-inflammatory cytokines IL-10 and arginase-1 for cell repair are produced/released from M2 phenotype microglia [43]. MeHg exposure increased pro-inflammatory factor expression without affecting anti-inflammatory factor expression, whereas gabapentin had no effect on the expression of these factors. Moreover, examination of phosphorylated CREB and BDNF, markers of neuronal activation [6, 49–55], showed that MeHg exposure increased neuronal activation in the spinal dorsal horn, but gabapentin treatment did not suppress this increase (Fig. 4).

TSPs have recently been demonstrated as important factors involved in synaptic rewiring that promotes excitatory synaptogenesis by acting on the alpha 2 delta-1 subunit of calcium channels [26, 27, 56]. Among others, TSP-1 is reportedly produced/released from glial cells in the somatosensory cortex upon nerve stimulation from the dorsal horn of the spinal cord, where it promotes the onset of neuropathic pain via synaptic rewiring with neuronal activation [28, 29]. In this study, MeHg exposure increased TSP-1 expression in the somatosensory region of the cerebral cortex, increasing the expression of synaptic components such as vGlut-1 and PSD-95 with neuronal activation, similar to our previous results [16, 17] (Fig. 5). Gabapentin had no effect on TSP-1 expression but showed an inhibitory effect on synaptogenesis and neuronal activation. Gabapentin exhibits antagonistic effects on TSP-1 at the alpha 2 delta-1 subunit of channels [26, 27]; thus, gabapentin likely suppressed synaptic rewiring through antagonism of its receptor without affecting TSP-1 expression. These results suggest that gabapentin reversibly modifies MeHg-induced *de* *novo* synaptogenesis in the somatosensory cortex, thereby suppressing neuronal activation and ultimately improving neuropathic pain. On the other hand, the fact that MeHg and Gabapentin did not affect synaptic rewiring in the spinal cord indicates that these effects are specific to the somatosensory cortex. In addition, mercury concentrations in the somatosensory cortex, thalamus, and spinal cord were unaffected by gabapentin administration (Fig. 6), indicating that gabapentin does not inhibit neuropathic pain by reducing the mercury concentration in cranial nerve tissue.

## Conclusions

This study is the first to demonstrate the alleviating effect of gabapentin on neuropathic pain in Minamata disease model rats. Furthermore, analysis of the mechanism suggests that the therapeutic effect of gabapentin might be mediated by reversibly modifying synaptic rewiring in the somatosensory cortex.

## Abbreviations

MeHg: Methylmercury
TNF: tumor necrosis factor
IL: interleukin
NF-κB: nuclear factor kappa B
CREB: CRE binding protein
iNOS: inducible nitric oxide synthase
TSP: thrombospondin
vGlut: vesicular glutamate transporter
PSD: postsynaptic density protein

## Supplementary information

**Additional file 1:** **Sup. Fig. 1** Histopathological analysis at the plantar part of the hind paw in Minamata disease model rats. Each image shows a representative image. (a) Hematoxylin and eosin staining at the center of the plantar part of the hind paw (bar = 100 µm). (b) Quantitative analysis of the distance from the skin surface to the border between the epidermis and dermis. Values represent the mean ± SEM (n = 6). (c) Body weight. Values are means ± SEM (n = 30∼6). Significant differences were observed between the vehicle treatment group and MeHg treatment groups (^#^p < 0.05 and ^##^p < 0.01).

## Declarations

### Availability of data and materials

The datasets generated and/or analyzed during the current study are available from the corresponding author upon reasonable request.

## References

[1] Takeuchi T. With special reference to its pathogenesis. Acta Pathologica Japonia: Pathology of Minamata Disease. 1982;32(1):73–99. 6765001

[2] Bakir F, Damluji SF, Amin-Zaki L, Murtadha M, Khalidi A, al-Rawi NY, Tikriti S, Dahahir HI, Clarkson TW, Smith JC, Doherty RA. Methylmercury poisoning in Iraq. Science. 1973;181(4096):230–41. 4719063 10.1126/science.181.4096.230

[3] Kurland LT, Faro SN, Siedler H. Minamata disease. The outbreak of a neurologic disorder in Minamata, Japan, and its relationship to the ingestion of seafood contaminated by mercuric compounds. World Neurol. 1960;1:370–95. 13755288

[4] Eto K. Pathology of Minamata disease. Toxicol Pathol. 1997;25(6):614–23. 9437807 10.1177/019262339702500612

[5] Eto K, Tokunaga H, Nagashima K, Takeuchi T. An autopsy case of Minamata disease (methylmercury poisoning) pathological viewpoints of peripheral nerves. Toxicol Pathol. 2002;30(6):714–22. 12512873 10.1080/01926230290166805

[6] Fujimura M, Usuki F. Site-specific neural hyperactivity via the activation of MAPK and PKA/CREB pathways triggers neuronal degeneration in methylmercury-intoxicated mice. Toxicol Lett. 2017 a;271:66–73. 28267559 10.1016/j.toxlet.2017.03.001

[7] Tohyama S, Usuki F. A fetal-type Minamata disease patient whose ADL was improved through decrease in plantar pain and spasticity produced by repeated vibratory therapy. Sogo Rehabilitation. 2011;39(11):1091–4.

[8] Usuki F, Tohyama S. Vibration therapy of the plantar fascia improves spasticity of the lower limbs of a patient with fetal-type Minamata disease in the chronic stage. BMJ Case Rep. 2011;bcr0820114695. 10.1136/bcr.08.2011.4695 PMC3207789 22675016

[9] Craig AD, Bushnell MC, Zhang ET, Blomqvist A. A thalamic nucleus specific for pain and temperature sensation. Nature. 1994;372(6508):770–3. 7695716 10.1038/372770a0

[10] Decosterd I, Woolf CJ. Spared nerve injury: an animal model of persistent peripheral neuropathic pain. Pain. 2000;87(2):149–58. 10924808 10.1016/S0304-3959(00)00276-1

[11] Klit H, Finnerup NB, Jensen TS. Central post-stroke pain: clinical characteristics, pathophysiology, and management. Lancet Neurol. 2009;8(9):857–68. 19679277 10.1016/S1474-4422(09)70176-0

[12] Shiao R, Lee-Kubli CA. Neuropathic Pain After Spinal Cord Injury: Challenges and Research Perspectives. Neurotherapeutics. 2018;15(3):635–53. 29736857 10.1007/s13311-018-0633-4 PMC6095789

[13] Echeverry S, Shi XQ, Zhang J. Characterization of cell proliferation in rat spinal cord following peripheral nerve injury and the relationship with neuropathic pain. Pain. 2008;135(1–2):37–47. 17560721 10.1016/j.pain.2007.05.002

[14] Henderson LA, Peck CC, Petersen ET, Rae CD, Youssef AM, Reeves JM, Gustin SM, . Chronic pain: lost inhibition? J Neurosci. 2013;33(17):7574–82. 23616562 10.1523/JNEUROSCI.0174-13.2013 PMC6619566

[15] Liang T, Chen XF, Yang Y, Yang F, Yu Y, Yang F, Chen J, . Secondary damage and neuroinflammation in the spinal dorsal horn mediate post-thalamic hemorrhagic stroke pain hypersensitivity: SDF1-CXCR4 signaling mediation. Front Mol Neurosci. 2022;15:911476. 36034499 10.3389/fnmol.2022.911476 PMC9416701

[16] Fujimura M, Usuki F, Nakamura A. Methylmercury induces hyperalgesia and allodynia through spinal cord dorsal horn neuronal activation and subsequent somatosensory cortical circuit formation in rats. Arch Toxicol. 2021;95(6):2151–62. 33847776 10.1007/s00204-021-03047-7

[17] Fujimura M. Fasudil, a ROCK inhibitor, prevents neuropathic pain in Minamata disease model rats. Toxicol Lett. 2022;371:38–45. 36244566 10.1016/j.toxlet.2022.10.001

[18] Goa KL, Sorkin EM. Gabapentin. A review of its pharmacological properties and clinical potential in epilepsy. Drugs. 1993;46(3):409–27. 7693432 10.2165/00003495-199346030-00007

[19] Bertrand S, Nouel D, Morin F, Nagy F, Lacaille JC. Gabapentin actions on Kir3 currents and N-type Ca2+ channels via GABAB receptors in hippocampal pyramidal cells. Synapse. 2003;50(2):95–109. 12923812 10.1002/syn.10247

[20] Tian GF, Azmi H, Takano T, Xu Q, Peng W, Lin J, Nedergaard M, . An astrocytic basis of epilepsy. Nat Med. 2005;11(9):973–81. 16116433 10.1038/nm1277 PMC1850946

[21] Bone M, Critchley P, Buggy DJ. Gabapentin in postamputation phantom limb pain: a randomized, double-blind, placebo-controlled, cross-over study. Reg Anesth Pain Med. 2002;27(5):481–6. 12373695 10.1053/rapm.2002.35169

[22] Smith DG, Ehde DM, Hanley MA, Campbell KM, Jensen MP, Hoffman AJ, Robinson LR. Efficacy of gabapentin in treating chronic phantom limb and residual limb pain. J Rehabil Res Dev. 2005;42(5):645–54. 16586190 10.1682/jrrd.2005.05.0082

[23] Coderre TJ, Kumar N, Lefebvre CD, Yu JS. Evidence that gabapentin reduces neuropathic pain by inhibiting the spinal release of glutamate. J Neurochem. 2005;94(4):1131–9. 16092950 10.1111/j.1471-4159.2005.03263.x

[24] Suzuki R, Rahman W, Rygh LJ, Webber M, Hunt SP, Dickenson AH. Spinal-supraspinal serotonergic circuits regulating neuropathic pain and its treatment with gabapentin. Pain. 2005;117(3):292–303. 16150546 10.1016/j.pain.2005.06.015

[25] Yang JL, Xu B, Li SS, Zhang WS, Xu H, Deng XM, Zhang YQ. Gabapentin reduces CX3CL1 signaling and blocks spinal microglial activation in monoarthritic rats. Mol Brain. 2012;5:18. 22647647 10.1186/1756-6606-5-18 PMC3517515

[26] Eroglu C, Allen NJ, Susman MW, O’Rourke NA, Park CY, Ozkan E, Barres BA, . Gabapentin receptor alpha2delta-1 is a neuronal thrombospondin receptor responsible for excitatory CNS synaptogenesis. Cell. 2009;139(2):380–92. 19818485 10.1016/j.cell.2009.09.025 PMC2791798

[27] Taylor CP, Harris EW. Analgesia with gabapentin and pregabalin may involve *N*-methyl-d-aspartate receptors, Neurexins, and Thrombospondins. J Pharmacol Exp Ther. 2020;374(1):161–74. 32321743 10.1124/jpet.120.266056

[28] Kim SK, Hayashi H, Ishikawa T, Shibata K, Shigetomi E, Shinozaki Y, Nabekura J, . Cortical astrocytes rewire somatosensory cortical circuits for peripheral neuropathic pain. J Clin Invest. 2016;126(5):1983–97. 27064281 10.1172/JCI82859 PMC4855913

[29] Danjo Y, Shigetomi E, Hirayama YJ, Kobayashi K, Ishikawa T, Fukazawa Y, Koizumi S, . Transient astrocytic mGluR5 expression drives synaptic plasticity and subsequent chronic pain in mice. J Exp Med. 2022;219(4):e20210989. 35319723 10.1084/jem.20210989 PMC8952801

[30] Fujimura M, Usuki F. *In situ* different antioxidative systems contribute to site-specific methylmercury neurotoxicity in mice. Toxicology. 2017 b;392:55–63. 29030019 10.1016/j.tox.2017.10.004

[31] Fujimura M, Usuki F, Unoki T. Decreased plasma thiol antioxidant capacity precedes neurological signs in a rat methylmercury intoxication model. Food Chem Toxicol. 2020 b;146:111810. 33058990 10.1016/j.fct.2020.111810

[32] Fujimura M, Usuki F. Pregnant rats exposed to low level methylmercury exhibit cerebellar synaptic and neuritic remodeling during the perinatal period. Arch Toxicol. 2020 a;94(4):1335–47. 32140736 10.1007/s00204-020-02696-4

[33] Fujimura M, Usuki F, Nakamura A. Fasudil, a ROCK inhibitor, recovers methylmercury-induced axonal degeneration by changing microglial phenotype in rats. Toxicol Sci. 2019;168(1):126–36. 30462329 10.1093/toxsci/kfy281

[34] Ito D, Imai Y, Ohsawa K, Nakajima K, Fukuuchi Y, Kohsaka S. Microglia-specific localisation of a novel calcium binding protein, Iba1. Brain Res Mol Brain Res. 1998;57(1):1–9. 9630473 10.1016/s0169-328x(98)00040-0

[35] Fujimura M, Usuki F. Low concentrations of methylmercury inhibit neural progenitor cell proliferation associated with up-regulation of glycogen synthase kinase 3β and subsequent degradation of cyclin E in rats. Toxicol Appl Pharmacol. 2015;288(1):19–25. 26184774 10.1016/j.taap.2015.07.006

[36] Fujimura M, Usuki F. Methylmercury induces oxidative stress and subsequent neural hyperactivity leading to cell death through the p38 MAPK-CREB pathway in differentiated SH-SY5Y cells. Neurotoxicology. 2018;67:226–33. 29913201 10.1016/j.neuro.2018.06.008

[37] Fujimura M, Usuki F, Cheng J, Zhao W. Prenatal low-dose methylmercury exposure impairs neurite outgrowth and synaptic protein expression and suppresses TrkA pathway activity and eEF1A1 expression in the rat cerebellum. Toxicol Appl Pharmacol. 2016;298:1–8. 26965727 10.1016/j.taap.2016.03.002

[38] Fujimura M, Cheng J, Zhao W. Perinatal exposure to low-dose methylmercury induces dysfunction of motor coordination with decreases in synaptophysin expression in the cerebellar granule cells of rats. Brain Res. 2012;1464:1–7. 22587888 10.1016/j.brainres.2012.05.012

[39] Mouraux A, Iannetti GD, Plaghki L. Low intensity intra-epidermal electrical stimulation can activate Aδ-nociceptors selectively. Pain. 2010;150(1):199–207. 20510515 10.1016/j.pain.2010.04.026

[40] Zimmerman A, Bai L, Ginty DD. The gentle touch receptors of mammalian skin. Science. 2014;346(6212):950–4. 25414303 10.1126/science.1254229 PMC4450345

[41] Backonja M, Glanzman RL. Gabapentin dosing for neuropathic pain: evidence from randomized, placebo-controlled clinical trials. Clin Ther. 2003;25(1):81–104. 12637113 10.1016/s0149-2918(03)90011-7

[42] Colburn RW, Rickman AJ, DeLeo JA. The effect of site and type of nerve injury on spinal glial activation and neuropathic pain behavior. Exp Neurol. 1999;157(2):289–304. 10364441 10.1006/exnr.1999.7065

[43] Tang Y, Le W. Differential roles of M1 and M2 microglia in neurodegenerative diseases. Mol Neurobiol. 2016;53(2):1181–94. 25598354 10.1007/s12035-014-9070-5

[44] Wolf SA, Boddeke HW, Kettenmann H. Microglia in physiology and disease. Annu Rev Physiol. 2017;79:619–43. 27959620 10.1146/annurev-physiol-022516-034406

[45] Jurga AM, Paleczna M, Kuter KZ. Overview of general and discriminating markers of differential microglia phenotypes. Front Cell Neurosci. 2020;14:198. 32848611 10.3389/fncel.2020.00198 PMC7424058

[46] Hanisch UK, Kettenmann H. Microglia: active sensor and versatile effector cells in the normal and pathologic brain. Nat Neurosci. 2007;10(11):1387–94. 17965659 10.1038/nn1997

[47] Iwai-Shimada M, Takahashi T, Kim MS, Fujimura M, Ito H, Toyama T, Hwang GW, . Methylmercury induces the expression of TNF-α selectively in the brain of mice. Sci Rep. 2016;6:38294. 27910896 10.1038/srep38294 PMC5133575

[48] Zhang X, Li W, Abudureheman A, Cheng T, Peng P. Imperatorin possesses notable anti-inflammatory activity in vitro and in vivo through inhibition of the NF-κB pathway. Mol Med Rep. 2017;16(6):8619–26. 28990061 10.3892/mmr.2017.7706 PMC5779915

[49] Valjent E, Caboche J, Vanhoutte P. Mitogen-activated protein kinase/extracellular signal-regulated kinase induced gene regulation in brain: a molecular substrate for learning and memory? Mol Neurobiol. 2001;23:83–99. 11817219 10.1385/MN:23:2-3:083

[50] Thomas GM, Huganir RL. MAPK cascade signalling and synaptic plasticity. Nat Rev Neurosci. 2004;5:173–83. 14976517 10.1038/nrn1346

[51] Chen Y, Fenoglio KA, Dubé CM, Grigoriadis DE, Baram TZ. Cellular and molecular mechanisms of hippocampal activation by acute stress are age-dependent. Mol Psychiatry. 2006;11(11):992–1002. 16801951 10.1038/sj.mp.4001863 PMC2927976

[52] Ji RR, Suter MR. p38 MAPK, microglial signaling, and neuropathic pain. Mol Pain. 2007;3:33. 17974036 10.1186/1744-8069-3-33 PMC2186318

[53] Boulle F, Massart R, Stragier E, Païzanis E, Zaidan L, Marday S, Lanfumey L, . Hippocampal and behavioral dysfunctions in a mouse model of environmental stress: normalization by agomelatine. Transl Psychiatry. 2014;4(11):e485. 25423137 10.1038/tp.2014.125 PMC4259995

[54] Walsh JJ, Friedman AK, Sun H, Heller EA, Ku SM, Juarez B, Burnham VL, Mazei-Robison MS, Ferguson D, Golden SA, Koo JW, Chaudhury D, Christoffel DJ, Pomeranz L, Friedman JM, Russo SJ, Nestler EJ, Han MH. Stress and CRF gate neural activation of BDNF in the mesolimbic reward pathway. Nat Neurosci. 2014;17:27–9. 24270188 10.1038/nn.3591 PMC3984932

[55] Jasińska KK, Molfese PJ, Kornilov SA, Mencl WE, Frost SJ, Lee M, Pugh K, Grigorenko EL, Landi N. The BDNF Val66Met polymorphism influences reading ability and patterns of neural activation in children. PLoS One. 2016;11:e0157449. 27551971 10.1371/journal.pone.0157449 PMC4995017

[56] Christopherson KS, Ullian EM, Stokes CC, Mullowney CE, Hell JW, Agah A, Barres BA, . Thrombospondins are astrocyte-secreted proteins that promote CNS synaptogenesis. Cell. 2005;120(3):421–33. 15707899 10.1016/j.cell.2004.12.020

