# Supplementary figures and images for: Gabapentin improves neuropathic pain in Minamata disease model rats
Source: Environ Health Prev Med. 2024 May 31;29:31. doi: 10.1265/ehpm.24-00035 (PMC11157338; doi:10.1265/ehpm.24-00035)

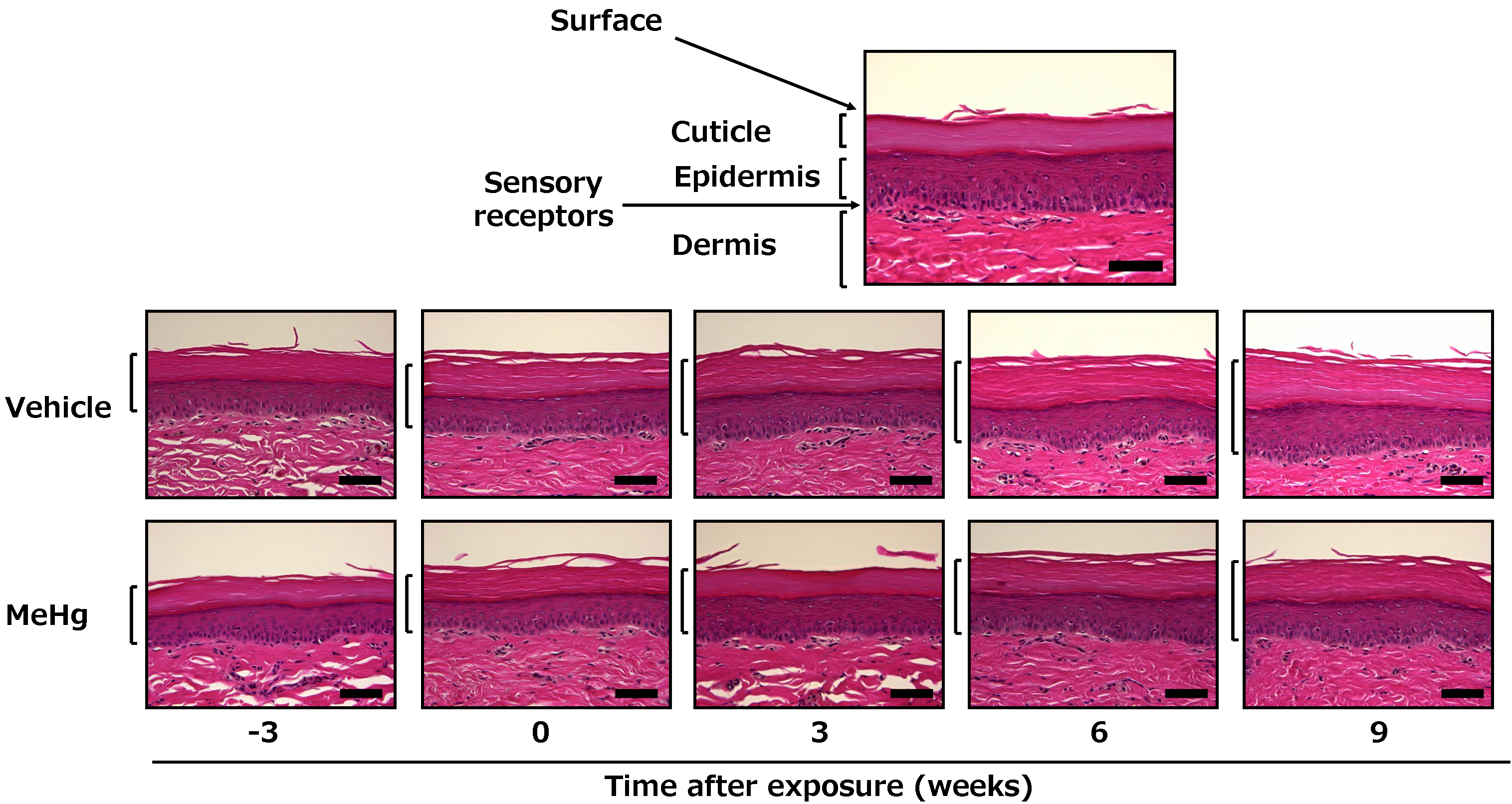

Supplement: Supplementary file 1 — Additional file 1: Sup. Fig. 1 Histopathological analysis at the plantar part of the hind paw in Minamata disease model rats. Each image shows a representative image. (a) Hematoxylin and eosin staining at the center of the plantar part of the hind paw (bar = 100 µm). (b) Quantitative analysis of the distance from the skin surface to the border between the epidermis and dermis. Values represent the mean ± SEM (n = 6). (c) Body weight. Values are means ± SEM (n = 30∼6). Significant differences were observed between the vehicle treatment group and MeHg treatment groups (#p < 0.05 and ##p < 0.01). [file ehpm-29-031-s001.zip › Sup.Fig.1a[EnvironHealthPreMed](MFujimura).tif]

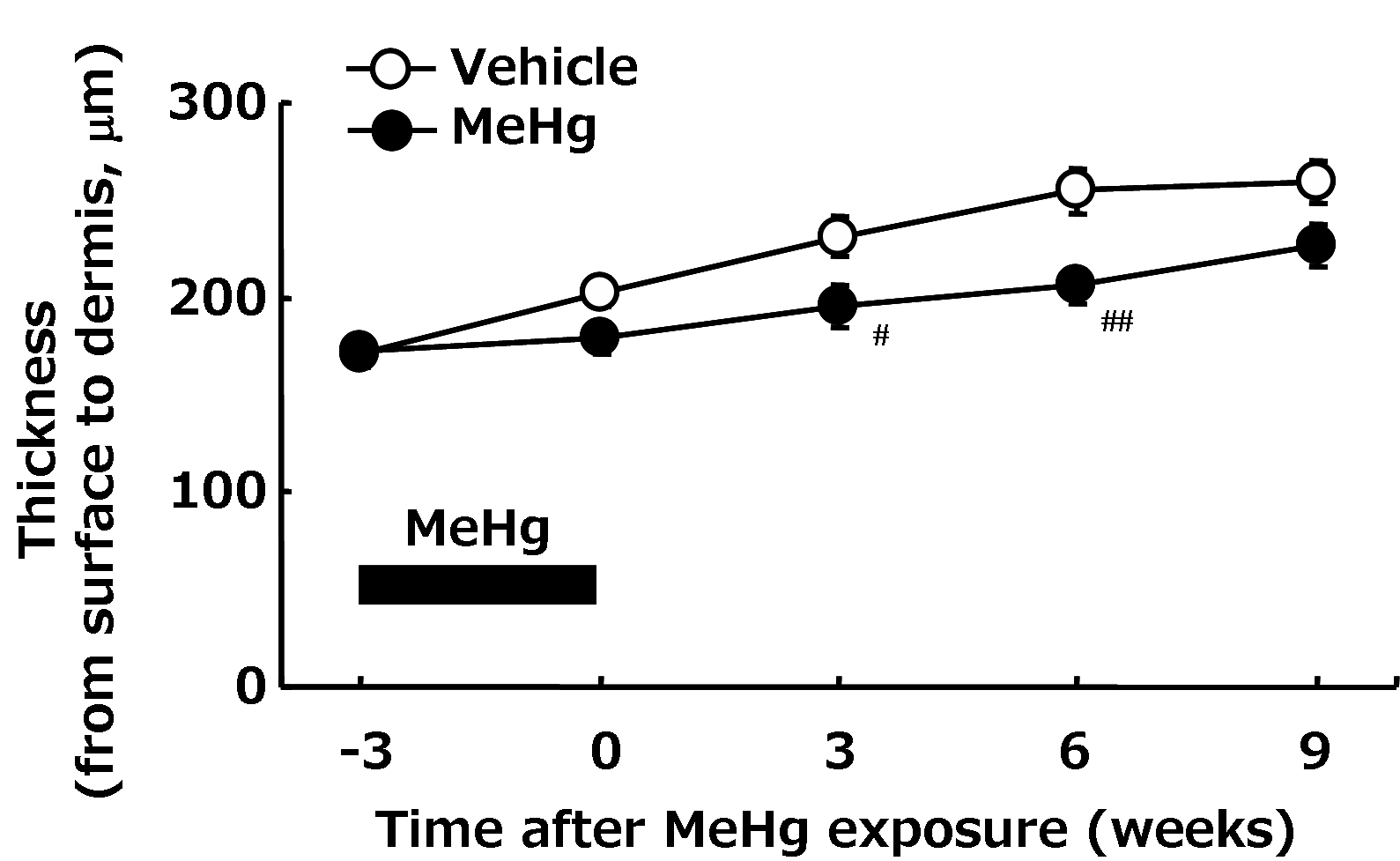

Supplement: Supplementary file 1 — Additional file 1: Sup. Fig. 1 Histopathological analysis at the plantar part of the hind paw in Minamata disease model rats. Each image shows a representative image. (a) Hematoxylin and eosin staining at the center of the plantar part of the hind paw (bar = 100 µm). (b) Quantitative analysis of the distance from the skin surface to the border between the epidermis and dermis. Values represent the mean ± SEM (n = 6). (c) Body weight. Values are means ± SEM (n = 30∼6). Significant differences were observed between the vehicle treatment group and MeHg treatment groups (#p < 0.05 and ##p < 0.01). [file ehpm-29-031-s001.zip › Sup.Fig.1b[EnvironHealthPreMed](MFujimura).tif]

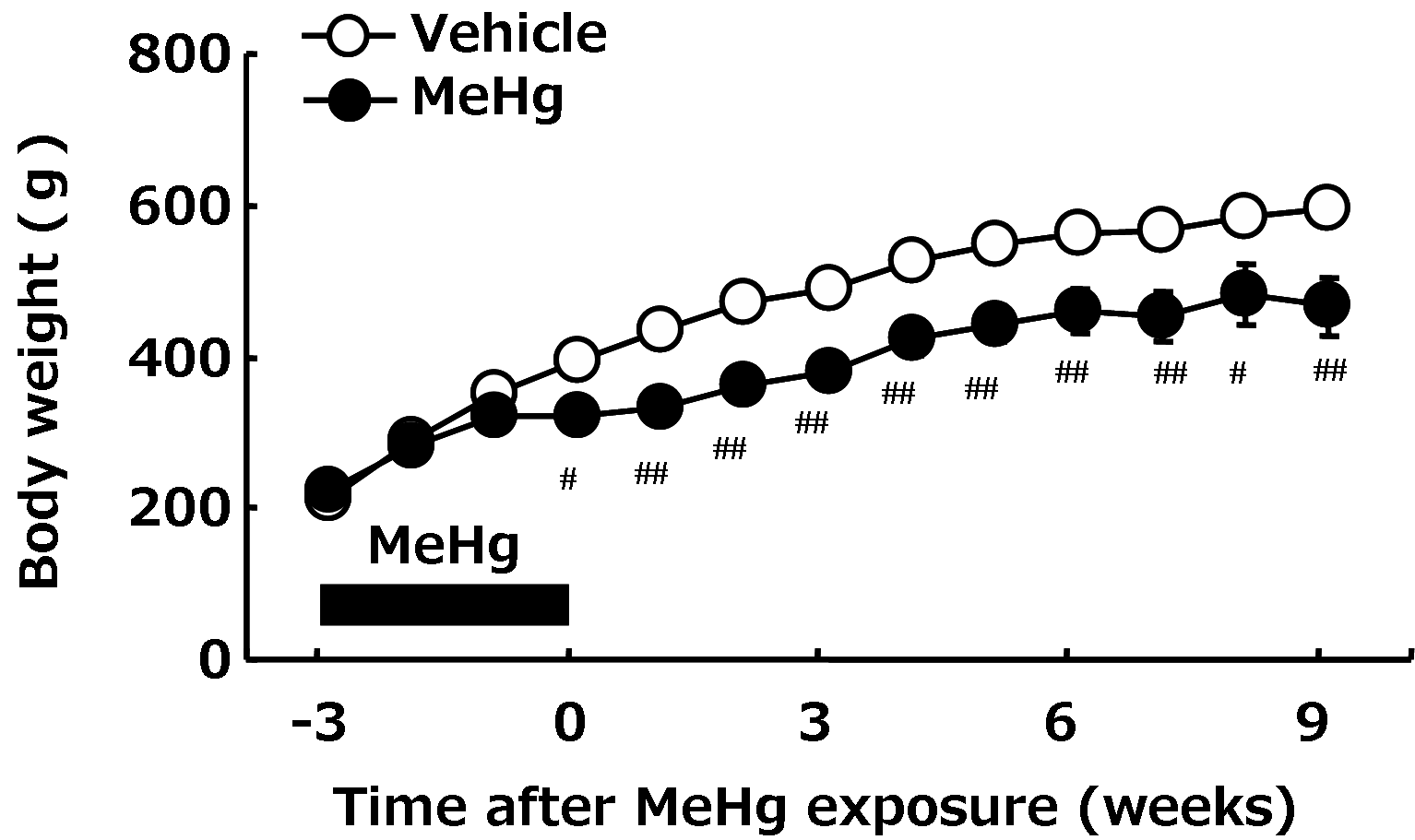

Supplement: Supplementary file 1 — Additional file 1: Sup. Fig. 1 Histopathological analysis at the plantar part of the hind paw in Minamata disease model rats. Each image shows a representative image. (a) Hematoxylin and eosin staining at the center of the plantar part of the hind paw (bar = 100 µm). (b) Quantitative analysis of the distance from the skin surface to the border between the epidermis and dermis. Values represent the mean ± SEM (n = 6). (c) Body weight. Values are means ± SEM (n = 30∼6). Significant differences were observed between the vehicle treatment group and MeHg treatment groups (#p < 0.05 and ##p < 0.01). [file ehpm-29-031-s001.zip › Sup.Fig.1c[EnvironHealthPreMed](MFujimura).tif]
